# HPV Status as Prognostic Biomarker in Head and Neck Cancer—Which Method Fits the Best for Outcome Prediction?

**DOI:** 10.3390/cancers13184730

**Published:** 2021-09-21

**Authors:** Jan Philipp Kühn, Wendelin Schmid, Sandrina Körner, Florian Bochen, Silke Wemmert, Hugo Rimbach, Sigrun Smola, Julia Caroline Radosa, Mathias Wagner, Luc G.T. Morris, Victoria Bozzato, Alessandro Bozzato, Bernhard Schick, Maximilian Linxweiler

**Affiliations:** 1 Department of Otorhinolaryngology, Saarland University Medical Center, D-66421 Homburg, Germany; jan.kuehn@uks.eu (J.P.K.); wendelin.schmid@uks.eu (W.S.); sandrina.koerner@uks.eu (S.K.); florian.bochen@uks.eu (F.B.); silke.wemmert@uks.eu (S.W.); hugo.rimbach@uks.eu (H.R.); Victoria.bozzato@uks.eu (V.B.); alessandro.bozzato@uks.eu (A.B.); bernhard.schick@uks.eu (B.S.); 2 Institute of Virology, Saarland University Medical Center, D-66421 Homburg, Germany; sigrun.smola@uks.eu; 3 Department of Gynecology and Obstetrics, Saarland University Medical Center, D-66421 Homburg, Germany; Julia.radosa@uks.eu; 4 Department of General and Surgical Pathology, Saarland University Medical Center, D-66421 Homburg, Germany; mathias.wagner@uks.eu; 5 Immunogenomics and Precision Oncology Platform, Memorial Sloan Kettering Cancer Center, New York, NY 10065, USA; morrisl@mskcc.org; 6 Department of Surgery, Memorial Sloan Kettering Cancer Center, New York, NY 10065, USA

**Keywords:** head and neck cancer, HPV, prognosis, biomarker, p16

## Abstract

**Simple Summary:**

Head and neck cancer belongs to the six most common cancers worldwide, with the majority of cases being associated with chronic alcohol and/or tobacco consumption. Over the past years, a rising proportion of those cancer cases are rather caused by a mucosal infection with high-risk human papillomavirus (HPV). In general, those patients are younger and respond better to different treatment options, which results in an overall better prognosis. Despite this high importance of HPV status in head and neck cancer, there is no established standard biomarker for the detection of HPV-related tumor biology. In our study, we tested six different detection methods that are currently used and compared their diagnostic and prognostic validity in a collective of 153 head and neck cancer patients. Thereby, immunohistochemical staining of p16—a protein biomarker that is overexpressed in HPV-related cancers—showed the best performance as prognostic biomarker and should be combined with a direct detection of HPV-DNA in case of therapeutic consequences.

**Abstract:**

The incidence of human papillomavirus (HPV)-related head and neck cancer (HNSCC) is rising globally, presenting challenges for optimized clinical management. To date, it remains unclear which biomarker best reflects HPV-driven carcinogenesis, a process that is associated with better therapeutic response and outcome compared to tobacco/alcohol-induced cancers. Six potential HPV surrogate biomarkers were analyzed using FFPE tissue samples from 153 HNSCC patients (*n* = 78 oropharyngeal cancer (OPSCC), *n* = 35 laryngeal cancer, *n* = 23 hypopharyngeal cancer, *n* = 17 oral cavity cancer): p16, CyclinD1, pRb, dual immunohistochemical staining of p16 and Ki67, HPV-DNA-PCR, and HPV-DNA-in situ hybridization (ISH). Biomarkers were analyzed for correlation with one another, tumor subsite, and patient survival. P16-IHC alone showed the best performance for discriminating between good (high expression) vs poor outcome (low expression; *p* = 0.0030) in OPSCC patients. Additionally, HPV-DNA-ISH (*p* = 0.0039), HPV-DNA-PCR (*p* = 0.0113), and p16-Ki67 dual stain (*p* = 0.0047) were significantly associated with prognosis in uni- and multivariable analysis for oropharyngeal cancer. In the non-OPSCC group, however, none of the aforementioned surrogate markers was prognostic. Taken together, P16-IHC as a single biomarker displays the best diagnostic accuracy for prognosis stratification in OPSCC patients with a direct detection of HPV-DNA by PCR or ISH as well as p16-Ki67 dual stain as potential alternatives.

## 1. Introduction

Head and neck squamous cell carcinomas (HNSCCs) belong to the six most common cancers worldwide and account for approximately 890,000 new cases and 450,000 deaths each year [1,2]. For many decades, alcohol and tobacco consumption were considered as the predominant risk factors for the development of this disease. In the 1990s, evidence began to emerge of a causal relationship between a subset of HNSCCs, especially those tumors arising from the oropharyngeal region, and mucosal infection with high-risk human papillomaviruses (HR-HPV) [3]. While around 7% of oropharyngeal squamous cell carcinomas (OPSCC) had evidence of HPV infection at the beginning of the 1990s, the prevalence of HPV-associated OPSCC markedly increased over the past decades [4]. To date mucosal HPV infection accounts for up to 41% of all OPSCCs in Europe and up to 70% of OPSCCs in the United States [5,6]. It is estimated that HPV-related head and neck cancers will surpass the number of HPV-related cervical cancers in the U.S. in the 2020s [7], which emphasizes the growing importance of this tumor entity with an urgent need for reliable diagnostic tools and effective therapeutic strategies [5]. 

In recent years, intensive research has uncovered essential mechanisms of pathogenesis and tumor biology in HPV-related OPSCC, and showed that HPV-induced HNSCCs are clinically and biologically distinct from tobacco- and alcohol-induced head and neck tumors [5]. Patients with HPV-related disease are younger, often not alcohol and tobacco consumers, frequently have clinically evident lymph node metastases at presentation, and respond better to radiotherapy and chemoradiation. Together, these factors have resulted in significantly improved overall survival among patients with HPV-associated oropharyngeal HNSCC, compared to HPV-negative tumors [8,9,10]. In contrast, in non-oropharyngeal head and neck cancer, the association between HPV status and therapeutic response and prognosis remains unclear, because there are no large, prospective studies [11]. Hence, HPV-status–especially in OPSCC patients–is an important prognostic and predictive biomarker, potentially enabling better prognostication and choice of therapeutic options. 

This clinically important differentiation between HPV-associated and HPV-independent OPSCC is mirrored in the 8th edition of UICC/AJCC staging system for head and neck cancers, which includes p16 immunostaining as a surrogate for HPV status in oropharyngeal cancer cases [12]. However, the use of p16 as a surrogate marker for HPV-driven OPSCCs is intensively discussed in literature since high p16 expression is often but not always an indicator of HPV-driven carcinogenesis [13], with a false-positive rate of 10–15% [14]. Especially with regard to the rising number of clinical trials evaluating de-escalated therapy regimens for HPV-associated OPSCC [15], the availability of highly reliable and accurate diagnostic tests identifying HPV-driven OPSCCs is a major unmet need. Various detection methods for HPV-associated disease are used in clinical practice, although they have unclear prognostic and predictive value. 

**Figure 1:**
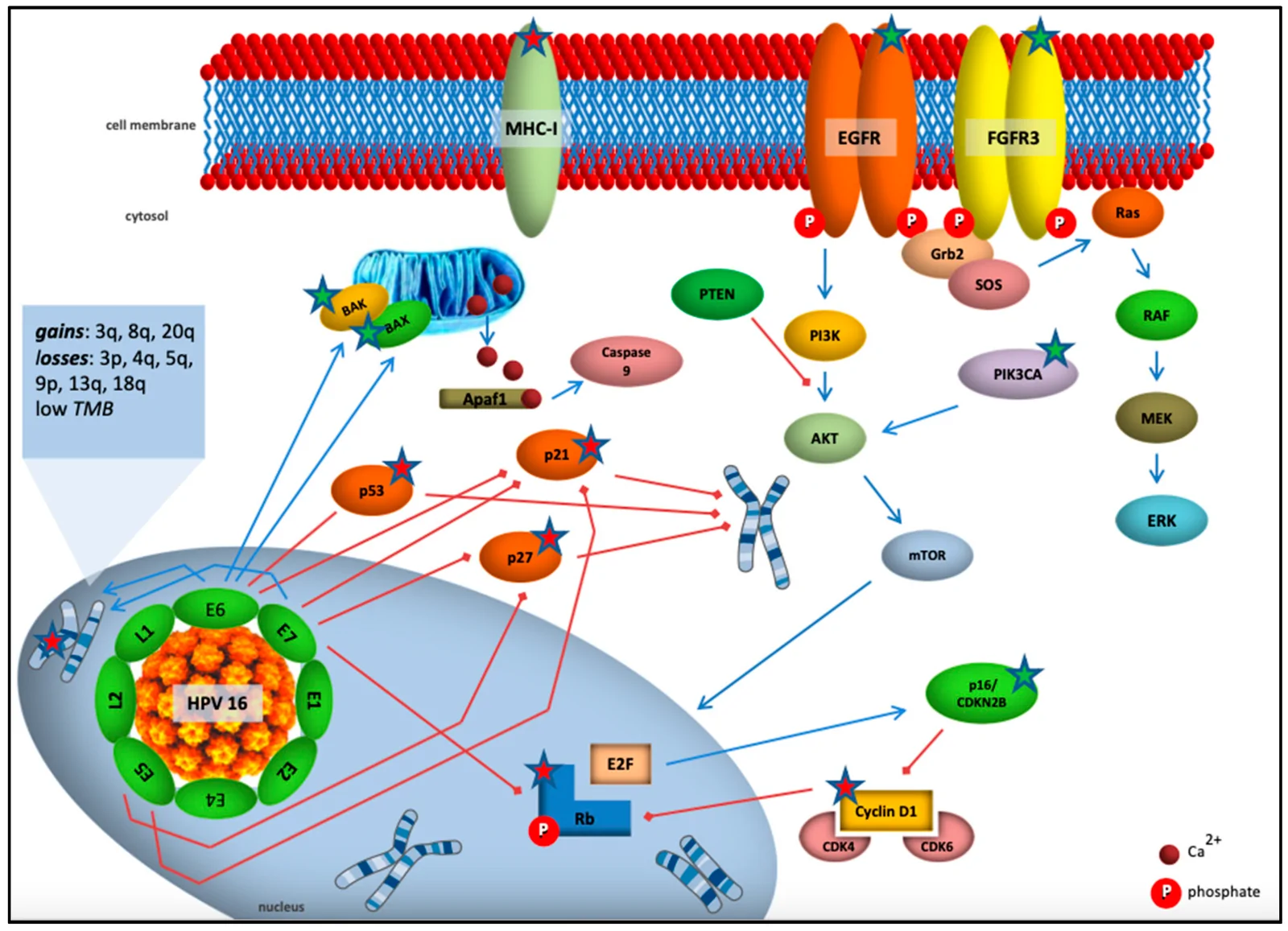
Tumor biology of HPV-driven head and neck squamous cell carcinomas. Increased expression levels are indicated by green stars, red stars indicate decreased expression levels. Blue arrows indicate pathway activation and/or increased expression, respectively. Red arrows indicate pathway inhibition and/or decreased expression, respectively. TMB—tumor mutational burden.

Based on the molecular biology of HPV-associated OPSCC (Figure 1), several potential biomarkers have been investigated in recent years for assessing HPV status: Direct detection of HPV-DNA either by polymerase chain reaction (PCR) or in-situ hybridization (ISH), detection of mRNA-transcripts of viral oncogenes E6 and E7, or immunohistochemical analysis of protein biomarkers whose expression level is regulated by E7 including p16, pRb, and CyclinD1. While the detection of E6- and E7-mRNA by reverse transcription PCR is currently considered the gold standard for reliably identifying HPV-driven OPSCC [16,17,18], this method is not feasible for widespread use in clinical practice due to high demands on sample quality and technical equipment. Ideally, alternative biomarkers must have both high sensitivity and specificity, offer cost-effective and logistically simple laboratory testing, and be amenable to uniform protocols across different laboratories and validation in prospective clinical trials. A surrogate marker meeting these requirements would potentially offer substantial value in clinical practice for guiding therapeutic decisions and assessing patients’ prognosis. With regard to limitations of the aforementioned potential HPV biomarkers [19,20,21,22,23], current data are ambiguous and the ideal, clinically tractable HPV surrogate marker for OPSCC still remains undefined.

Therefore, we compared the prognostic relevance of six practically applicable biomarkers for assessing HPV status in 153 HNSCC patients, including HPV-DNA-PCR, HPV-DNA-ISH, immunohistochemical p16-Ki67 dual staining, and the protein expression of p16, CyclinD1, and pRb. Those biomarkers were correlated with patients’ survival data in uni- and multivariable analyses and compared between different anatomical subsites. 

## 2. Materials and Methods

### 2.1. Patient Characteristics and Tissue Samples

In total, 153 patients were enrolled in this study with histopathological diagnosis of head and neck squamous cell carcinomas of the oropharynx (*n* = 78), larynx (*n* = 35), hypopharynx (*n* = 23), and oral cavity (*n* = 17). The patient cohort comprised 125 male (125/153; 82%) and 28 female patients (28/153; 18%). The mean age of the patients was 64 years. All patients were treated at the Department of Otorhinolaryngology, Head and Neck Surgery at the Saarland University Medical Center (Homburg, Germany). Further clinical characteristics including TNM and UICC stages are shown in Table 1. For all included patients, we used the 7th version of the UICC TNM classification as the majority of patients analyzed in this study was diagnosed before 2017.

None of the patients presented with distant metastasis. Written informed consent was obtained from all patients. The Saarland Medical Association ethics review committee approved the scientific use of the patients’ tissue and clinical data.

### 2.2. Immunohistochemistry

For each of the 153 included HNSCC patients, representative formalin-fixed paraffin-embedded (FFPE) tissue samples of the primary tumor were obtained for further immunohistochemical analysis. All 153 tumor samples were pathologically classified as moderately (G2; *n* = 91) to poorly differentiated (G3; *n* = 62) squamous cell carcinomas. In addition, hematoxylin and eosin (HE) stains were generated for each sample for morphological control using a standard protocol. After omitting the first three 10 μm sections of the FFPE-block, consecutive 4 μm sections were prepared, transferred onto Superfrost Ultra Plus microscope slides (Menzel-Gläser, Braunschweig, Germany), and dried in an incubator at 37 °C overnight. Upon deparaffinization, heat-induced epitope retrieval was performed in 10 mM citrate buffer (pH 6.0) and unspecific protein binding sites were blocked by incubation in 200 mL PBS (Phosphate-buffered saline, Sigma Aldrich, St. Louis, MO, USA) and 6 g bovine serum albumin (BSA, Sigma Aldrich, St. Louis, MO, USA) under pH control (7.2) for 30 min at room temperature. Afterwards, immunohistochemical staining was performed targeting p16, p16, and Ki67 simultaneously, pRb, and CyclinD1. For immunohistochemical dual stain of p16 and Ki67, the CINtec PLUS kit was used applying a modified manufacturer protocol as described in our previous work [24]. For all other target proteins, the primary antibody incubation was performed for 1 h at room temperature with a 1:4000 dilution in 1% BSA/PBS for p16 ^INK4a^ (ab54210; abcam, Cambridge, UK), a 1:700 dilution in 1% BSA/PBS for pRb (ab181616; abcam, Cambridge, UK), and a 1:125 dilution in 1% BSA/PBS for CyclinD1 (ab16663; abcam, Cambridge, UK). Controls included a positive control for each staining series as well as a negative control by omission of the primary antibody. Visualization was then performed via Streptavidin Alkaline Phosphatase and Chromogen Red using the Dako REAL™ Detection System Alkaline Phosphatase/RED (Dako GmbH, Glostrup, Denmark) according to the manufacturer’s instructions. Finally, slides were counterstained with hematoxylin and mounted with Entellan^®^ new Medium (Merck KGaA, Darmstadt, Germany). 

The immunoreactivity for p16 and p16-Ki67, pRb and CyclinD1 was independently evaluated by four examiners: one pathologist, one human biologist, one otorhinolaryngologist and one technical assistant, all with wide experience in evaluating immunohistochemical staining. For p16-Ki67 dual staining, a positive result was only assigned when ≥50% of the tumors cells showed a dual expression of Ki67 (nuclear signal) and p16 (cytoplasmic signal). For p16, pRb, and CyclinD1 immunoreactivity was evaluated using an immunoreactive score (IRS) according to Remmele and Stegner [25]. Hereby, the percentage of positively stained cells is rated from 0 to 4 and staining intensity is rated from 0 to 3. Multiplication of both values results in the final IRS ranging from 0 (no immunoreactivity) to 12 (strong immunoreactivity). 

### 2.3. HPV-DNA In Situ Hybridization

For the detection of HPV-DNA in FFPE-tissue-slides by in situ hybridization (ISH), the Ventana INFORM^®^ HPV III Family 16 probe (Roche Diagnostics GmbH, Mannheim, Germany) was used according to the manufacturer’s instructions targeting the common high-risk HPV genotypes found to be associated with human malignancies including HPV 16, 18, 31, 33, 35, 39, 45, 51, 52, 56, 58, and 66. For the visualization of labeled DNA, the Ventana ISH iView Blue Plus detection kit (Roche Diagnostics GmbH, Mannheim, Germany) was used according to the manufacturer’s instructions. HPV-DNA-ISH was performed for all 153 HNSCC patients. HPV-DNA-ISH was evaluated by an experienced pathologist and rates as positive when at least 50% of the lesional cells showed distinct nuclear and/or cytoplasmic signals. 

### 2.4. HPV-DNA-PCR

HPV-DNA specific PCR was performed for all patients using FFPE-tissue specimens. DNA was extracted from the FFPE-tissue samples using the QIAamp DNA FFPE Tissue Kit (Qiagen *n*.V., Hilden, Germany) according to the manufacturer’s instructions. HPV-PCR was performed with the LightCycler 2.0 instrument (Roche Diagnostics GmbH, Mannheim, Germany) using the GP5+/6+ primers as described by De Roda Husman et al. [26] that can detect at least 27 mucosotropic HPV types including HPV 6, 11, 16, 18, 31 and 33. SYBR green as well as gel electrophoresis were used for detection. After initial denaturation at 95 °C for 15 min, 45 PCR cycles followed with denaturation at 95 °C for 10 s, annealing at 45 °C for 5 s, and elongation at 72 °C for 18 s. After amplification, a melting curve was performed at temperatures between 45 and 95 °C, with temperature increasing at a rate of 0.2 °K s^−1^. The melting temperature for the HPV16-positive control was 79 and 82 °C for the HPV18-positive control. Glyceraldehyde 3-phosphate dehydrogenase (GAPDH) PCR was performed in parallel for each sample as a positive control, as described by Ruprecht et al. [27]. 

### 2.5. Statistical Analysis

GraphPad Prism 9.0 (GraphPad Software, La Jolla, CA, USA) and SPSS Version 27 (IBM, Ehningen, Germany) were used for statistical analysis presuming a significance level of 5% (α = 0.05) and a statistical power of 80% (β = 0.8). The existence of normal distribution for the analyzed biomarkers p16, CyclinD1, and pRb was controlled by Kolmogorov–Smirnov test, Anderson–Darling test, D’Agostino–Pearson test, and Shapiro–Wilk test. Homogenous variance was checked by Levine test. If parameters showed no normal distribution in at least one of the aforementioned tests, non-parametric Mann–Whitney U test was used. In case of normal distribution in all of the aforementioned tests, a two-sided t-test was used. For survival analyses the Kaplan–Meier algorithm and the log-rank test were used. Correlation analyses between two dichotomous variables were performed using a Fisher’s exact test. Correlation between two continuous variables was calculated by Spearman’s correlation analysis. Odd’s ratios (OR) with 95% confidence intervals in multivariable analyses were calculated according to logistic regression with dummy coding for independent variables. For determining how well the data fit in the selected model corrected Akaike’s information criterion (cAIC) were calculated, with a smaller cAIC indicating a better model fit. *p*-values are indicated in the figures.

## 3. Results

### 3.1. Prognostic Associations of HPV Surrogate Markers

To evaluate the prognostic value of the six HPV surrogate markers, overall survival (OS) was correlated with biomarker expression (i.e., HPV-DNA-PCR (positive vs. negative), HPV-DNA-ISH (positive vs. negative), p16-Ki67 dual stain (positive vs. negative), p16-IRS (IRS ≥ 7 vs. IRS < 7), Cyclin D1-IRS (IRS ≥ 7 vs. IRS < 7), and pRb-IRS (IRS ≥ 7 vs. IRS < 7)). As it was shown that the prognostic relevance of HPV status is different depending on the anatomical subsite of the tumor, Kaplan–Meier analyses were performed separately for cancers arising from the oropharynx (*n* = 78), larynx (*n* = 35), hypopharynx (*n* = 23), and oral cavity (*n* = 17). Additionally, analyses were performed for three latter subsites grouped together as non-OPSCC. 

**Figure 2:**
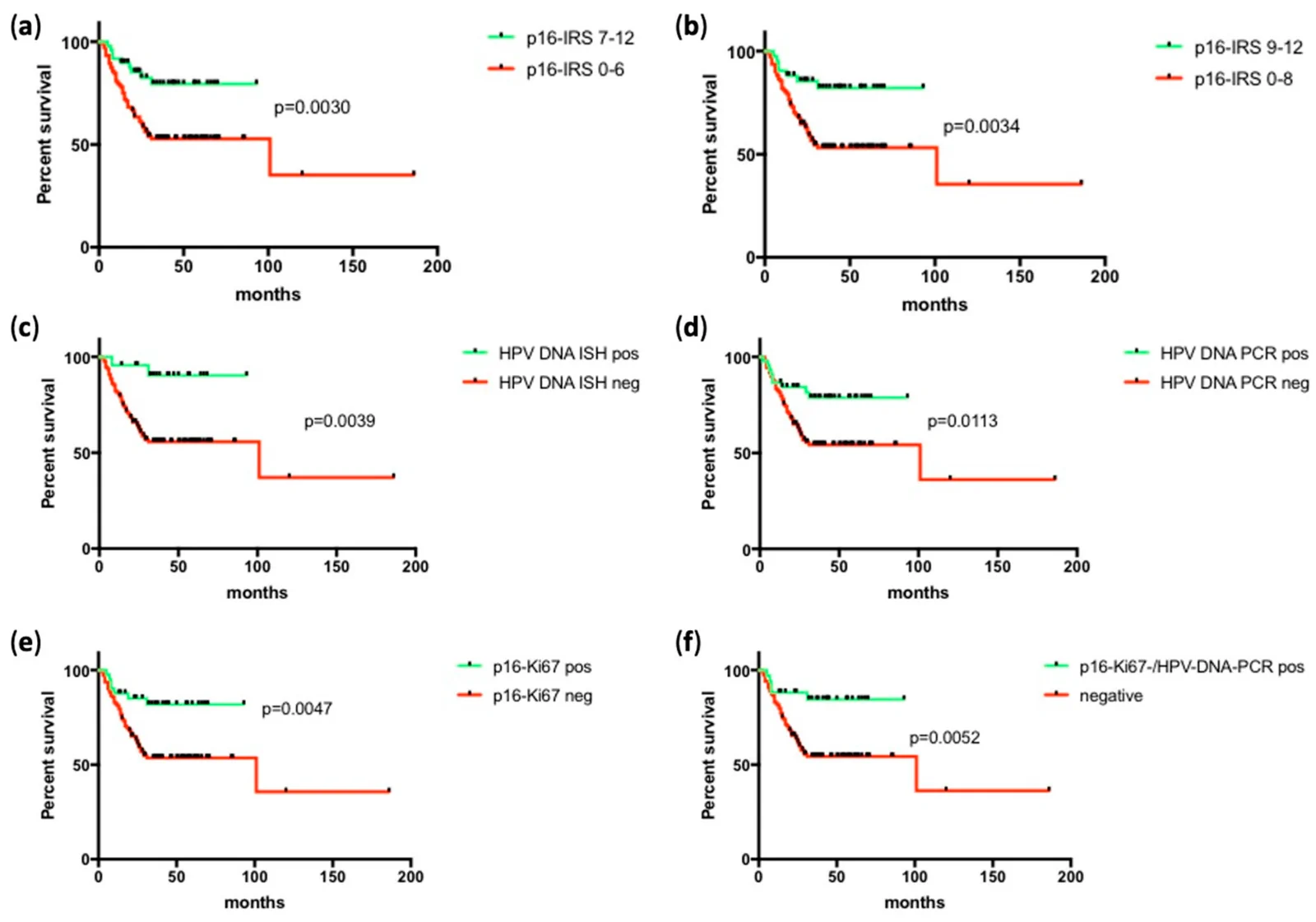
Overall survival of OPSCC patients depending on the detection of HPV-DNA by PCR or ISH and p16 expression. (**a**) p16 positive (green, IRS 7-12) vs. negative (red, IRS 0-6) patients; (**b**) patients grouped to p16-IRS 9-12 (high, green) and p16-IRS 0-8 (low, red); (**c**) HPV-DNA-ISH positive (green) vs. negative (red) patients; (**d**) HPV-DNA-PCR positive (green) vs. negative (red) patients; (**e**) p16-Ki67 dual positive (green) vs. negative (red) patients; (**f**) p16-Ki67 dual positive and HPV-DNA-PCR positive (green) vs. patients negative for p16-Ki67 dual staining and/or HPV-DNA-PCR (red). In (**a**)–(**f**), censored data are marked with a black dot. *p*-values are indicated for each graph (log-rank test). IRS—immunoreactive score.

For oropharyngeal cancer cases, p16 showed the highest significance in outcome prediction with a favorable OS for p16-high (IRS ≥ 7) compared to p16-low (IRS < 7) cases (*p* = 0.0030; Figure 2a). 

**Figure 3:**
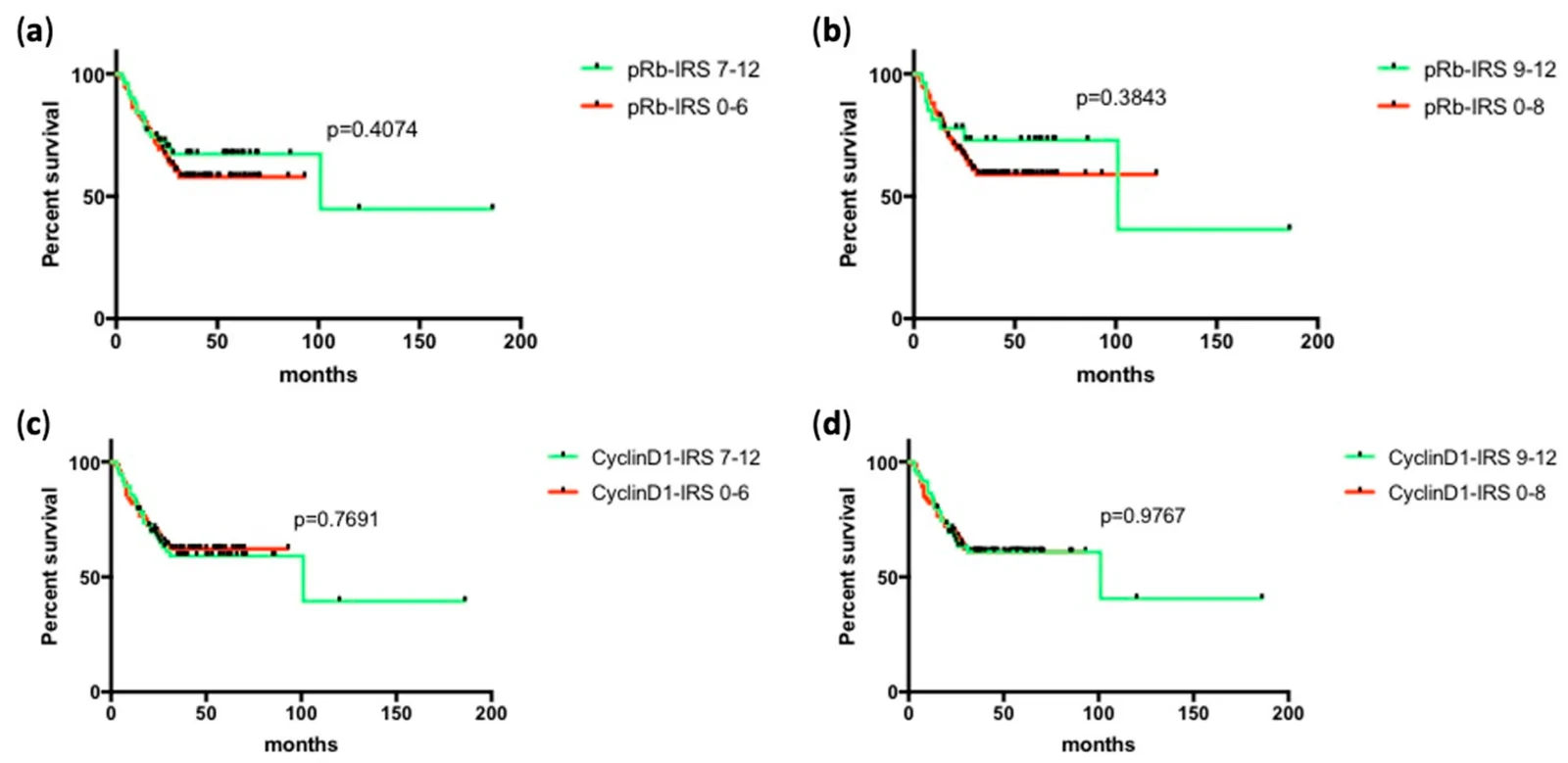
Overall survival of OPSCC patients depending on the expression of pRb and CyclinD1. (**a**) patients with high pRb expression (green, IRS 7-12) vs. low pRb expression (red, IRS 0-6) patients; (**b**) comparison of patients with pRb-IRS 9-12 (green) vs. pRb-IRS 0-8 (red); (**c**) patients with high CyclinD1 expression (green, IRS 7-12) vs. low CyclinD1 expression (red, IRS 0-6); (**d**) comparison of patients with CyclinD1-IRS 9-12 (green) vs. CyclinD1-IRS 0-8 (red). In (**a**)–(**d**), censored data are marked with a black dot. *p*-values are indicated for each graph (log-rank test). IRS–immunoreactive score.

Alternative IRS thresholds for defining p16-positive and p16-negative cases could not further improve the prognostic performance of p16 (example shown in Figure 2b for IRS ≥ 9 vs. IRS < 9). In addition, HPV-DNA-ISH and HPV-DNA-PCR positivity were predictors of significantly longer OS with, however, a slightly lower significance level compared to p16 (*p* = 0.0039 for HPV-DNA-ISH, Figure 2c; *p* = 0.0113 for HPV-DNA-PCR, Figure 2d). Positive p16-Ki67 dual staining of lesional cells was associated with a significantly better outcome compared to negative dual staining results, as shown in Figure 2e (*p* = 0.0047). For both pRb and CyclinD1 expression, we found no significant difference in survival time depending on protein expression level regardless of the IRS threshold for defining positive vs. negative cases (Figure 3). In addition, we tested all possible combinations of two biomarkers for the OPSCC group trying to further improve the significance of outcome prediction. However, none of these combinations reached a higher significance level than p16-IRS alone (exemplarily shown in Figure 2f for the combination of p16-Ki67 dual stain with HPV-DNA-PCR).

When extending the Kaplan–Meier analyses to the three non-OPSCC groups (laryngeal cancer, hypopharyngeal cancer, oral cavity cancer), we found no significant correlation of any of the aforementioned biomarkers with the patients’ overall survival, neither for the three localizations grouped together nor for any subgroup separately. 

To see if for the OPSCC cases p16 expression, HPV-DNA-ISH, HPV-DNA-PCR, and p16-Ki67 dual stain are also independent predictors of patient survival, we performed multivariable analyses for factors associated with overall survival benefit using logistic regression models including patient age, UICC cancer stage, and smoking status (≥10 pack years vs. <10 pack years) as covariates. P16 positivity (i.e., IRS ≥ 7; OR = 2.897; 95% CI 1.254–7.172), HPV-DNA-PCR positivity (OR = 2.383; 95% CI 1.100–6.162), HPV-DNA-ISH positivity (OR = 6.126; 95% CI 1.536–41.22), and p16-Ki67-dual-stain positivity (OR = 3.423; 95% CI 1.358–9.565) each independently predicted survival probability. CyclinD1-IRS and pRb-IRS did not show independent prognostic significance. For determining how well the data fit the selected model in multivariant logistic regression analyses, we calculated corrected Akaike’s information criterion (cAIC) and found the lowest value for HPV-DNA-ISH (189.5), followed by p16-Ki67 dual stain (188.8), p16-IHC (189.5), HPV-DNA-PCR (192.1), pRb-IHC (194.4), and CyclinD1-IHC (195.7). 

Beyond HPV surrogate markers, higher UICC stage (OR = 0.634; 95% CI 0.4048–0.9473) and a smoking status of ≥10 pack years (OR = 0.3566; 95% CI 0.1434–0.8446) were independent predictors of a worse outcome. Patients’ age showed no influence on overall survival (OR = 0.9846; 95% CI 0.9451-1.025). 

### 3.2. Correlation of HPV Surrogate Markers with Each Other

**Figure 4:**
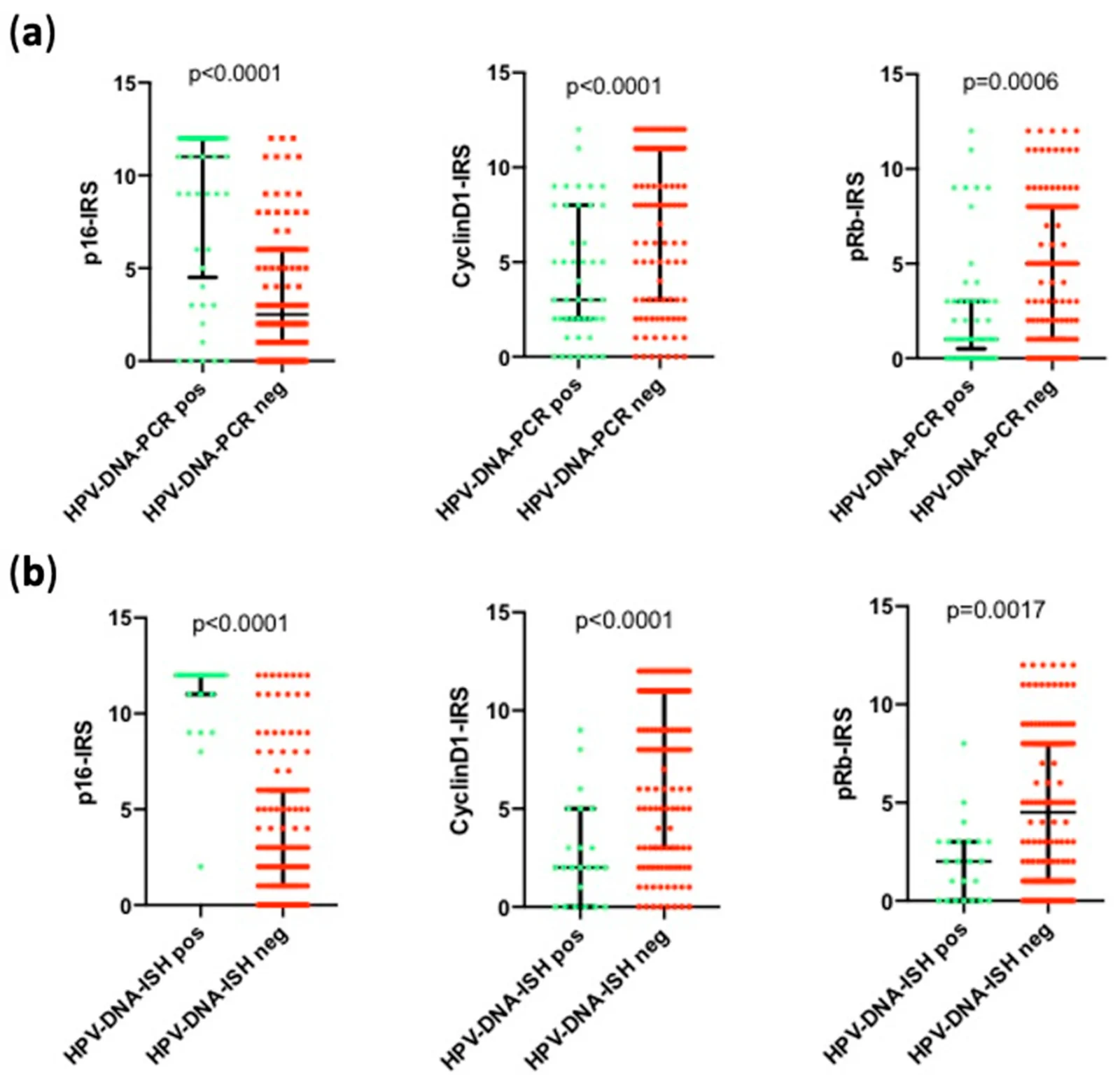
Expression of p16, CaclinD1, and pRb depending on the presence of HPV-DNA-PCR. (**a**) Expression of p16, CyclinD1, and pRb in HPV-DNA-PCR positive (green) vs. negative (red) patients. (**b**) Expression of p16, CyclinD1, and pRb in HPV-DNA-ISH positive (green) vs. negative (red) patients. In (**a**,**b**), each dot represents one case and the median is indicated by a line. The bars indicated the range from the first quartile to the third quartile. *p*-values are indicated for each graph (Mann–Whitney U test). IRS—immunoreactive score.

Based on the existing knowledge about the molecular biology of head and neck cancers associated with high-risk HPV infection, one would expect HPV-driven tumors to show positive reactions in HPV-DNA-PCR, HPV-DNA-ISH, and p16-Ki67 dual stain, as well as high expression levels for p16 with low expression levels of Cyclin D1 and pRb. Accordingly, we found significantly higher p16, Cyclin D1, and pRb protein levels in HPV-DNA-PCR positive cases and HPV-DNA-ISH positive cases compared to cases without detection of HPV-DNA by either method (*p* < 0.0001 for each protein biomarker, respectively; Mann–Whitney U test, Figure 4). Similarly, we found a strong correlation of HPV-DNA-ISH positivity with HPV-DNA-PCR positivity and p16-Ki67 positivity, as well as a strong correlation of HPV-DNA-PCR positivity with a positive p16-Ki67 dual stain (*p* < 0.0001 for all aforementioned comparisons, respectively; Fisher’s exact test; Supplementary Figure S1). 

**Figure 5:**
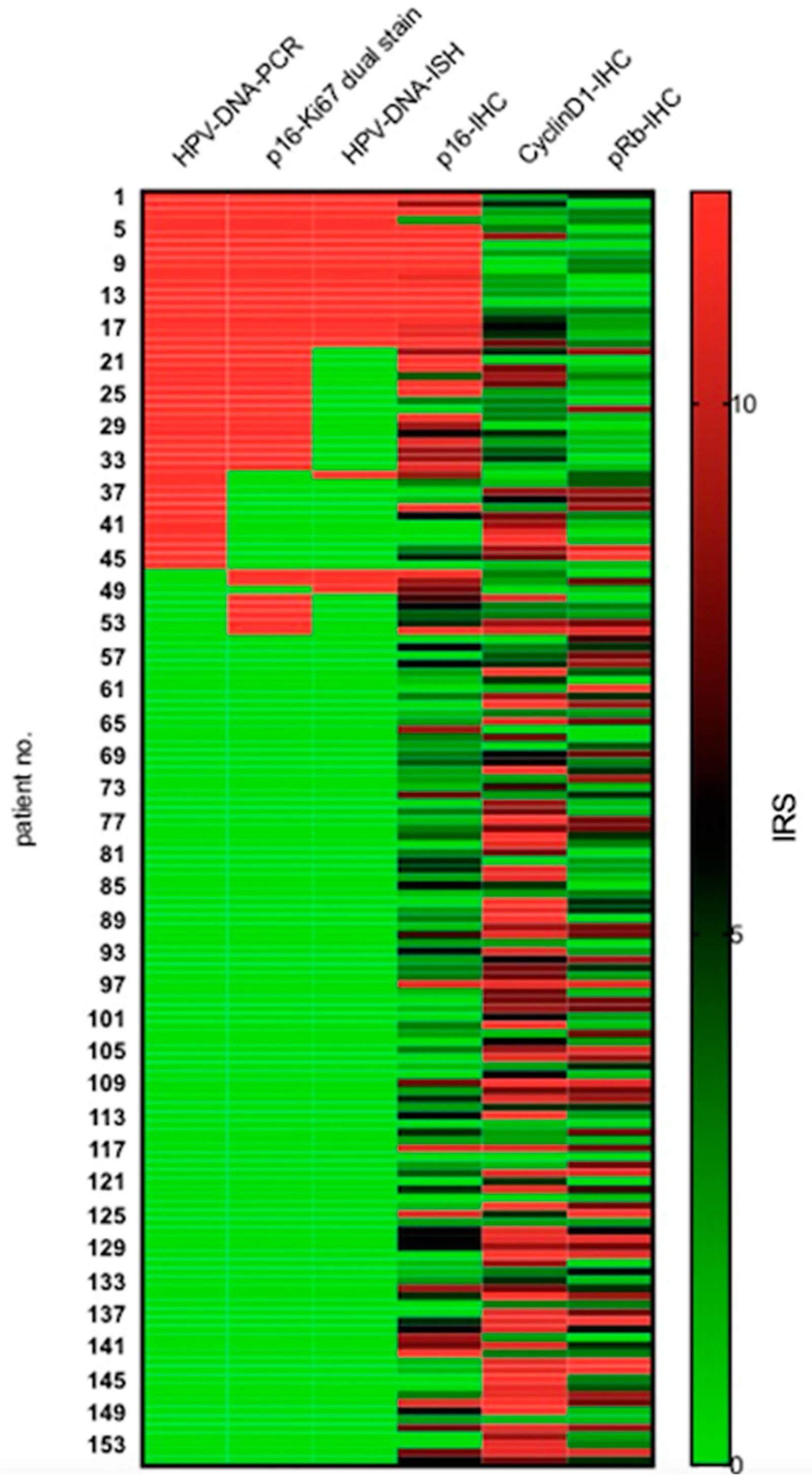
Illustration of all six investigated HPV surrogate markers delineated for the included 153 HNSCC patients in a heatmap. For HPV-DNA-PCR, p16-Ki67 dual stain, and HPV-DNA-ISH, positive testing results are indicated by a red line and negative testing results are indicated by a green line. For p16-, CyclinD1-, and pRb-IHC, the color of the lines ranges from light green (IRS 0) to light red (IRS12), as indicated by the scale bar on the right. IRS—immunoreactive score.

However, we found an expression pattern of the six investigated HPV surrogate markers only for a low number of cases that clearly indicates either HPV-driven disease or HPV-independent disease; however, rather a wide gray zone leaving space for interpretation. Figure 5 illustrates the results for all included patients and all investigated HPV surrogate markers in a heatmap. While HPV-DNA-PCR positivity showed a good correlation with HPV-DNA-ISH positivity, p16-Ki67 positivity, high p16 expression levels, and low Cyclin D1 expression levels in the majority of cases, pRb displayed no clear expression pattern with only a slight tendency towards lower expression levels in HPV-DNA positive cases.

### 3.3. HPV Surrogate Marker Expression Depending on Anatomical Subsite

**Figure 6:**
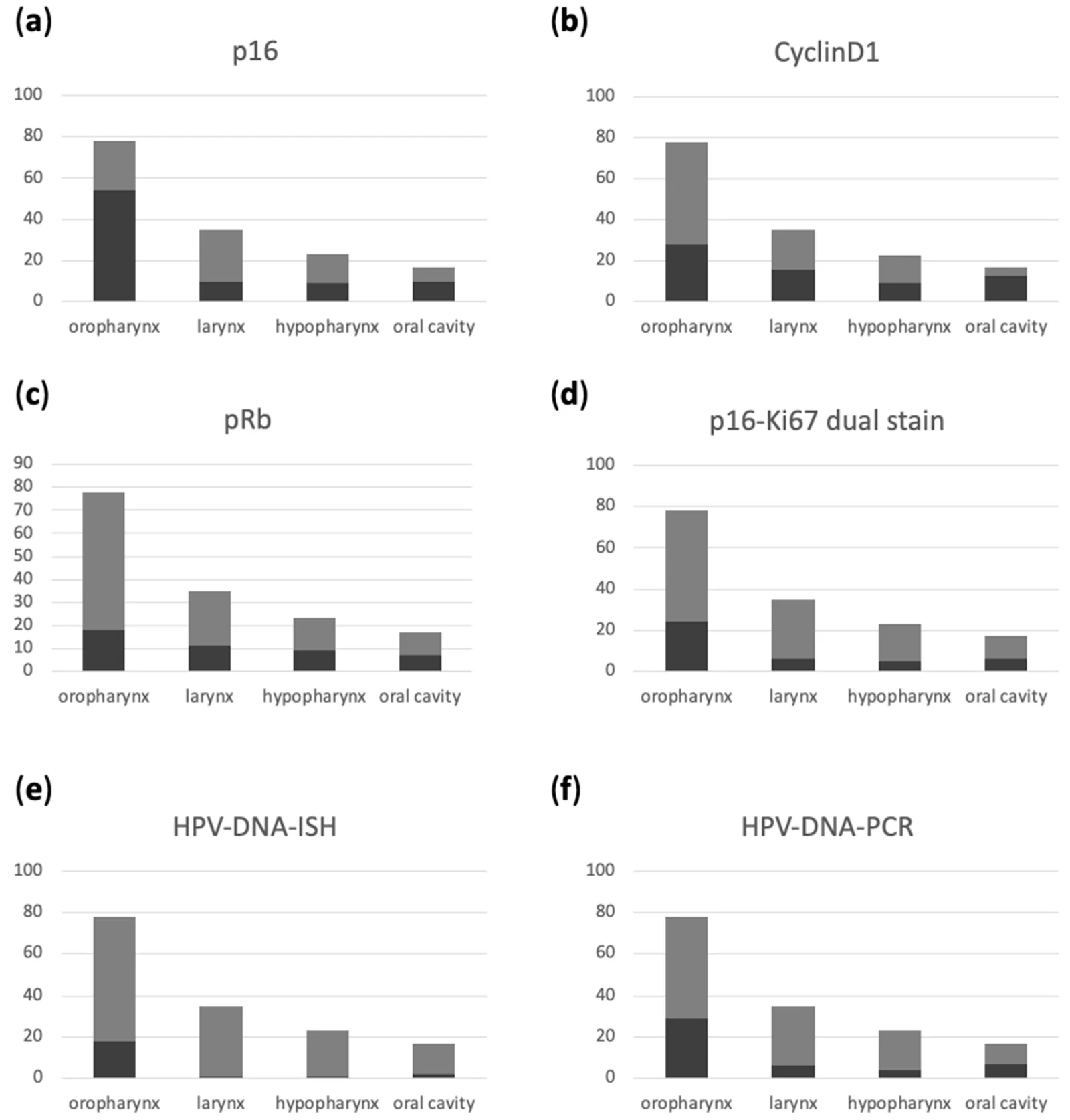
Expression pattern of HPV surrogate markers depending on anatomical localization of the primary tumor. Expression of p16 (**a**), Cyclin D1 (**b**), pRb (**c**), and results of p16-Ki67 dual stain (**d**), HPV-DNA-ISH (**e**), and HPV-DNA-PCR (**f**) delineated for oropharyngeal cancer (*n* = 78), laryngeal cancer (*n* = 35), hypopharyngeal cancer (*n* = 23), and cancer of the oral cavity (*n* = 17). In (**a**–**c**), immunohistochemical staining results with an IRS ≥ 7 are shown in black and staining results with an IRS < 7 are shown in gray. In (**d**–**f**), positive results are shown in black and negative results are shown in gray.

In the next step, we analyzed the expression of HPV surrogate markers depending on anatomical localization of the primary tumor. Thereby, oropharyngeal cancers showed the highest percentage of p16-positive cases (54%, 42/78, defined as p16-IRS ≥ 7), followed by oral cavity cancers (47%, 8/17), hypopharyngeal cancers (39%, 9/23), and laryngeal cancers (34%, 12/35; Figure 6). 

Comparable distributions were found for HPV-DNA-ISH (oropharynx = 23% positive cases, 18/78; oral cavity = 12% positive cases, 2/17; hypopharynx = 4% positive cases, 1/23; larynx = 3% positive cases, 1/35). For HPV-DNA-PCR and p16-Ki67 dual staining, most positive cases were found in cancers of the oral cavity (HPV-DNA-PCR = 41% positive cases, 7/17; p16-Ki67 dual stain = 35%, 6/17) followed by the oropharynx (HPV-DNA-PCR = 37% positive cases, 29/78; p16-Ki67 dual stain = 31%, 24/78), hypopharynx (HPV-DNA-PCR = 17% positive cases, 4/23; p16-Ki67 dual stain = 22%, 5/23), and larynx (HPV-DNA-PCR = 17% positive cases, 6/35; p16-Ki67 dual stain = 17%, 6/35). The highest percentage of Cyclin D1 positives cases (defined as CyclinD1 IRS ≥ 7) was observed in oral cavity cancer (77%, 13/17) with fewer positive cases of laryngeal cancer (46%, 16/35), hypopharyngeal cancer (39%, 9/23), and oropharyngeal cancer (36%, 28/78). Comparably, most pRb positive cases were found in cancers of the oral cavity (41%, 7/17), hypopharynx (39%, 9/23), larynx (31%, 11/35), and oropharynx (23%, 18/78). 

## 4. Discussion

In our study, we analyzed six potential HPV surrogate markers in FFPE-samples of 153 HNSCC patients and evaluated their prognostic relevance by uni- and multivariable tests of association with prognosis. We found that p16 showed the best performance in segregating a good vs. poor prognostic group. HPV-DNA-ISH, HPV-DNA-PCR, and p16-Ki67 dual staining were also significantly associated with survival in uni- and multivariable analyses. CyclinD1 and pRb were not prognostic. Despite clear tendencies of expression patterns when comparing the six different biomarkers with each other, a large gray zone was observed with ambiguous interpretation on the role of HPV in the individual tumor biology. 

Large-scale randomized prospective clinical trials of the past decades have provided strong evidence for the prognostic and predictive relevance of high-risk HPV in head and neck squamous cell carcinomas. HPV-associated tumors are associated with better response to radiotherapy and chemoradiotherapy, as well as improved overall survival, compared to tobacco-/alcohol-associated disease [28]. Additionally, smoking status and AJCC/UICC stage can further differentiate between low-risk cancer and intermediate-risk cancer within the HPV positive group of patients [9]. However, neither currently available clinical guidelines in the U.S. [29] nor in Europe [30] recommend a modification of established treatment regimens based on HPV status. Due to the dramatically rising incidence of HPV related disease worldwide and the relevant survival benefit associated with HPV positivity, more and more long-term survivors will have to face irreversible toxicities associated with high-intense primary or adjuvant RT/CRT including xerostomia, dysphagia, fatigue, polyneuropathy, and speaking difficulties over decades. This has motivated a number of de-escalation trials with different de-intensification strategies and study protocols aiming to achieve non-inferior oncological outcomes with improved quality of life in HPV positive HNSCC patients. However, recently published results of two large-scale phase-III de-escalation trials were rather disappointing: The RTOG-1016 trial included 805 p16+ oropharyngeal cancer patients and compared two treatment strategies in a non-inferiority approach: radiotherapy plus cetuximab vs. radiotherapy plus cisplatin [31]. The results of this study indicated that radiotherapy plus cetuximab was inferior to radiotherapy plus cisplatin with regard to overall survival and locoregional failure. In a comparable study design, the De-ESCALaTE HPV trial assigned a total of 334 patients with locally advanced low-risk p16+ oropharyngeal cancer to either radiotherapy plus cetuximab or radiotherapy plus cisplatin as primary treatment. Final results showed a significantly shorter overall survival as well as a higher rate of recurrences in the cetuximab arm, which clearly argues against the replacement of cisplatin with cetuximab as de-escalation strategy [32]. Strikingly, both studies only used p16-IHC to define HPV-related disease as an inclusion criterion, which is in line with current recommendations on HPV testing in oropharyngeal cancer patients according to guidelines of ESMO/ESTRO/EHNS [30] as well as College of American Pathologists [33]. However, this testing strategy bears the risk of a potential selection bias as several retrospective studies focusing on the outcome of HNSCC patients based on p16 expression have shown that there is in fact a 10–15% rate of false positive results when only relying on p16 as surrogate marker for HPV-related disease. For patients with high p16 expression but HPV-DNA negativity, p16 indicates a superior clinical outcome, but, in fact, those patients’ survival curves show more or less the same trends as alcohol- and tobacco-related disease [14,34]. It is possible that, if a different definition of HPV-positivity was used, the results of these pivotal de-escalation trials may have been different. Nevertheless, these data indicate that we must bear in mind the highly relevant risk of undertreatment, at least for a subset of patients, when offering de-escalated therapy regimens to HNSCC patients only based on p16 expression. 

Though our study showed no improved prognostic discrimination of OPSCC patients when comparing p16 alone with p16 + HPV-DNA-PCR or HPV-DNA-ISH as stratification biomarkers, we highly recommend using p16-IHC in combination with a direct detection of HPV-DNA whenever considering patients for de-escalated protocols. When solely focusing on the patients’ prognosis, p16 alone seems to be a robust biomarker and represents a good compromise, between acceptable diagnostic accuracy and easy applicability for everyday clinical practice, based on the results of our study, where p16 showed the best performance as a prognostic biomarker within all tested surrogate markers. 

With regard to the other biomarkers investigated in our study, HPV DNA PCR alone was shown not to be sufficiently accurate at identifying HPV-driven HNSCC due to laboratory contamination or detection of viral DNA depositions from other sites [19]. With regard to this, the HPV DNA in situ hybridization is superior, since the DNA is detected directly in the tumor cells. However, the disadvantage of this method is its low sensitivity [21]. These literature data are in line with our results: We found a markedly higher percentage of HPV-DNA-PCR+ compared to HPV-DNA-ISH+ HNSCC cases, with nearly all ISH+ cases being also PCR+, which indicates a favorable sensitivity for the PCR method and a favorable specificity for the ISH method. In addition to a direct detection of HPV-DNA and p16, there are several other proteins whose expression level is regulated by viral oncogene E7 and thus could serve as HPV surrogate markers including pRb and CyclinD1 [20,22,23]. In our study, CyclinD1 and pRb were not found to bear prognostic relevance in OPSCC patients. Similarly, Kumar et al. showed no correlation of CyclinD1 expression with tumor grade, stage of disease, and lymph node metastasis in OPSCC patients [35]. Plath et al. also showed that p16 alone is a reliable marker for survival prognostication in surgically treated OPSCC patients and that neither CyclinD1 nor pRb can further increase its prognostic performance [36]. In a study by Ribeiro et al. pRb turned out to be neither a reliable biomarker for the patients’ prognosis nor to predict recurrence in squamous cell carcinomas of the oropharynx [37]. One possible explanation for this lack of prognostic and predictive validity of CyclinD1 and pRb in spite of their close connection with HPV-E7 action (see Figure 1) may be that both proteins are embedded in a complex molecular network of protein–protein interaction, so that other pathways can modify their expression level as well [38,39]. 

Taken together, these data show that a direct detection of HPV-DNA either using PCR or ISH can reliably be used as HPV surrogate biomarkers in OPSCC patients with substantial prognostic validity. In contrast, CyclinD1 and pRb do not appear suitable for prognostication in OPSCC due to their limited correlation with HPV-driven tumor biology. 

From a critical point of view, one has to question why an additional direct testing of HPV-DNA either using PCR or ISH could not improve prognostic stratification in our OPSCC collective of patients. A possible explanation is that p16 itself has a relevant prognostic validity, independent of the molecular link to HPV-E7, due to its role as a tumor suppressor gene. In addition, within HPV-DNA negative patients, high p16 expression is associated with a relevant survival benefit [34]. In this context, one has to bear in mind that p16 expression is not exclusively regulated by HPV oncogene E7 but also influenced by other molecular mechanisms, including cellular senescence and tumor differentiation [40]. Hence, p16 cannot be regarded as a perfect surrogate for HPV infection but has to be seen as part of a complex regulatory network of a cell cycle independent of HPV. Hence, it is comprehensible that, though direct detection of HPV-DNA either by PCR or ISH in addition to p16-IHC presumably increases specificity for identifying the truly HPV-driven HNSCC cases, prognostication will not automatically become more precise. 

However, as improved response to radiation and chemoradiation is directly linked to HPV-driven cancer biology in OPSCC patients [41], we recommend a combination of p16-IHC and HPV-DNA detection for clinical practice whenever considering to draw therapeutic consequences out of HPV-status. In this scenario, the avoidance of undertreatment has a higher priority than an exact prognostication. 

Another potential weakness of our study is the relatively small number of patients especially in the non-OPSCC group, which markedly limits the validity of the Kaplan–Meier analyses for the laryngeal, hypopharyngeal, and oral cavity cancer patients. However, many other studies have shown that HPV and its surrogate markers are only prognostically relevant for tumors arising from the oropharynx and not for other tumor localizations [42,43,44,45,46], which is in line with our findings. As few other studies showed an influence of HPV on the prognosis and therapy response of non-OPSCC cancers [47,48,49] and large-scale, prospective clinical studies on that issue are still missing [11], we decided to include both OPSCC and non-OPSCC in our study cohort. Furthermore, the widely accepted diagnostic gold standard for detection of HPV-associated tumor disease (i.e., RT-PCR for detection of E6/E7-mRNA) was not carried out on our study samples, thus we cannot definitely say which tumors are truly HPV-independent and which tumors are HPV-driven. However, it was rather the aim of our study to investigate individual tumor biology in molecular detail and to focus on diagnostic methods that are easily applicable, practicable in clinical routine, and reliable for predicting patients’ prognosis. 

## 5. Conclusions

Taken together, our study showed that p16-IHC as a single biomarker displays the best diagnostic accuracy for prognosis stratification in oropharyngeal cancer patients, with high expression levels indicating a survival benefit. No combination with other biomarkers linked to HPV biology could further improve the performance of p16 as a prognostic biomarker, so that p16-IHC represents a good compromise between diagnostic accuracy and easy applicability for everyday clinical practice. However, one should add direct testing for HPV-DNA in p16-positive cases whenever therapeutic consequences are considered based on HPV status. Hence, the choice of HPV status biomarker should be driven by the clinical scenario: When focusing on the evaluation of prognosis, p16 alone is sufficient, whereas in the context of therapy stratification, a direct detection of HPV-DNA should be added. The highly diverse expression patterns of HPV-related biomarkers, as shown in this study, reveal that there is only a low fraction of clearly HPV-positive and clearly HPV-negative head and neck cancers, with a large gray zone in between. To assign those patients to diversified treatment regimens in order to avoid long-term toxicities and at the same time guarantee the best possible oncological outcome will be a major challenge for the clinical management of OPSCC in the future, and has to be addressed thoroughly in prospective clinical trials. 

## Figures and Tables

**Table 1:** Clinical characteristics of included HNSCC patients.

		HNSCC
Number of Patients		153
Sex	male female	125 28
Age ([years]; median, range)		64 (34–87)
Localization	oral cavity oropharynx hypopharynx larynx	17 78 23 35
T-stage	T1 T2 T3 T4	26 62 34 31
N-stage	N0 N1 N2b N3	40 26 81 6
UICC-stage	1 2 3 4a 4b 4c	12 17 31 81 5 7

## Data Availability

The data presented in this study are available on request from the corresponding author.

## Supplementary Materials

The following are available online at https://www.mdpi.com/article/10.3390/cancers13184730/s1, Figure S1: Correlation of HPV-DNA-PCR, HPV-DNA-ISH, and p16-Ki67 dual staining results.
