# Correction: Kühn et al. HPV Status as Prognostic Biomarker in Head and Neck Cancer—Which Method Fits the Best for Outcome Prediction? *Cancers* 2021, *13*, 4730

**DOI:** 10.3390/cancers18203291

**Published:** 2026-10-13

**Authors:** Jan Philipp Kühn, Wendelin Schmid, Sandrina Körner, Florian Bochen, Silke Wemmert, Hugo Rimbach, Sigrun Smola, Julia Caroline Radosa, Mathias Wagner, Luc G. T. Morris, Victoria Bozzato, Alessandro Bozzato, Bernhard Schick, Maximilian Linxweiler

**Affiliations:** 1 Department of Otorhinolaryngology, Saarland University Medical Center, D-66421 Homburg, Germany; jan.kuehn@uks.eu (J.P.K.); wendelin.schmid@uks.eu (W.S.); sandrina.koerner@uks.eu (S.K.); florian.bochen@uks.eu (F.B.); silke.wemmert@uks.eu (S.W.); hugo.rimbach@slk-kliniken.de (H.R.); victoria.bozzato@uks.eu (V.B.); alessandro.bozzato@uks.eu (A.B.); bernhard.schick@uks.eu (B.S.); 2 Institute of Virology, Saarland University Medical Center, D-66421 Homburg, Germany; sigrun.smola@uks.eu; 3 Department of Gynecology and Obstetrics, Saarland University Medical Center, D-66421 Homburg, Germany; julia.radosa@uks.eu; 4 Department of General and Surgical Pathology, Saarland University Medical Center, D-66421 Homburg, Germany; 5 Immunogenomics and Precision Oncology Platform, Memorial Sloan Kettering Cancer Center, New York, NY 10065, USA; morrisl@mskcc.org; 6 Department of Surgery, Memorial Sloan Kettering Cancer Center, New York, NY 10065, USA

## Text Correction

There was an error in the original publication [1].

Original text:

“For all other target proteins, the primary antibody incubation was performed for 1 h at room temperature with a 1:4000 dilution in 1% BSA/PBS for p16 ^INK4a^ (ab51243; abcam, Cambridge, UK), a 1:700 dilution in 1% BSA/PBS for pRb (ab181616; abcam, Cambridge, UK), and a 1:125 dilution in 1% BSA/PBS for CyclinD1 (ab16663; abcam, Cambride, UK).”

A correction has been made to Section 2.2 Immunohistochemistry; the corrected text is as follows:

“For all other target proteins, the primary antibody incubation was performed for 1 h at room temperature with a 1:4000 dilution in 1% BSA/PBS for p16 ^INK4a^ (ab54210; abcam, Cambridge, UK), a 1:700 dilution in 1% BSA/PBS for pRb (ab181616; abcam, Cambridge, UK), and a 1:125 dilution in 1% BSA/PBS for CyclinD1 (ab16663; abcam, Cambridge, UK).”

The authors state that the scientific conclusions are unaffected. This correction was approved by the Academic Editor. The original publication has also been updated.
