# Supplementary material for: HPV Status as Prognostic Biomarker in Head and Neck Cancer—Which Method Fits the Best for Outcome Prediction?
Source: Cancers (Basel). 2021 Sep 21;13(18):4730. doi: 10.3390/cancers13184730 (PMC8469433; doi:10.3390/cancers13184730)
Supplement: Supplementary file 1 [file cancers-13-04730-s001.zip › cancers-1363757-supplementary.pdf]

Fisher's exact test

Data analyzed	p16-Ki67 pos	p16-Ki67 neg	Total
HPV-DNA-PCR pos	34	11	45
HPV-DNA-PCR neg	7	101	108
Total	41	112	153

P<0.0001

Data analyzed	HPV-DNA-ISH pos	HPV-DNA-ISH neg	Total
HPV-DNA-PCR pos	20	25	45
HPV-DNA-PCR neg	3	105	108
Total	23	130	153

P<0.0001

Data analyzed	p16-Ki67 pos	p16-Ki67 neg	Total
HPV-DNA-ISH pos	21	2	23
HPV-DNA-ISH neg	20	110	130
Total	41	112	153

P<0.0001

Supplementary Figure S1: Correlation of HPV-DNA-PCR, HPV-DNA-ISH, and p16-Ki67 dual staining results. Results of HPV-DNA-PCR, HPV-DNA-ISH, and p16-Ki67 dual stain are shown in contingency tables. Correlation between HPV-DNA-PCR and p16-Ki67 dual stain (upper table), HPV-DNA-PCR and HPV-DNA-ISH (middle table) as well as HPV-DNA-ISH and p16-Ki67 dual stain (lower table) was analyzed using Fisher's exact test.
